# Editorial: Rumen microbiome: interacting with host genetics, dietary nutrients metabolism, animal production, and environment

**DOI:** 10.3389/fmicb.2023.1267149

**Published:** 2023-09-14

**Authors:** Qingbiao Xu, Emilio M. Ungerfeld, Diego P. Morgavi, Sinead M. Waters, Jinxin Liu, Wenjuan Du, Shengguo Zhao

**Affiliations:** ^1^College of Animal Sciences and Technology, Huazhong Agricultural University, Wuhan, China; ^2^Centro Regional de Investigación Carillanca, Instituto de Investigaciones Agropecuarias, Vilcún, La Araucanía, Chile; ^3^INRAE, VetAgro Sup, UMR Herbivores, Université Clermont Auvergne, Saint-Genès-Champanelle, France; ^4^Teagasc, Animal and Bioscience Research Department, Animal and Grassland Research and Innovation Centre, Dunsany, Ireland; ^5^College of Animal Science and Technology, Nanjing Agricultural University, Nanjing, China; ^6^Institute of Animal Sciences, Chinese Academy of Agricultural Sciences, Beijing, China

**Keywords:** rumen microbiome, nutrition, host, environment, animal production

Ruminants play an essential role in global human societies due to their unique ability to convert low-value feeds into high quality protein food (meat and milk) for human consumption using their rumen microbiome. The rumen microbiome is the ruminant's second genome, which facilitates the transformation of feeds into valuable animal products. The rumen microbiome profoundly influences dietary nutrient metabolism, animal production, the quality of animal products, and the environment. Understanding the complex interactions among microbial groups and with the host is essential to optimizing rumen function to meet growing demands for animal products while minimizing the environmental impact of ruminant production.

The aim of this Research Topic is to improve our understanding of the rumen microbiota interactions and fermentation control as the key to developing interventions toward sustainable intensification of ruminant production. The 14 publications gathered in this Research Topic have expanded our knowledge of the role of the rumen microbiome.

Cattle and other ruminants produce large amounts of the potent greenhouse gas methane. Enteric methane is a natural byproduct of microbial fermentation of feed in the rumen or gastrointestinal tract, accounts for 6% of total emissions of CH4 from anthropogenic related sources (Difford et al., 2018). The rumen microbiome, along with the host, influences methane emissions and feed efficiency of dairy cows. The papers by Malik et al. and O'Hara et al. reported the effects of the addition of anti-methanogenic products on the rumen microbiomes of sheep and dairy cows, respectively. Malik et al. reported that the addition of 5% of a tannin-containing supplements to a straw and concentrate mixed diet resulted in significantly reduced enteric methane emissions from sheep without affecting intake and rumen fermentation. The anti-methanogenic supplementation had no effect on the core microbiome, but significantly reduced rumen protozoa and reduced the activity of key enzymes involved in rumen methanogenesis. O'Hara et al. evaluated the effects of three red seaweeds on rumen prokaryotic communities *in vitro*. The population of rumen methanogens collapsed almost completely after supplementation with *Asparagopsis taxiformis*, and the number of methanogens that recovered during the stabilization phase was small. Similarly, *A. taxiformis* inhibited bacteria involved in acetate and propionate synthesis and fiber degradation, thereby reducing volatile fatty acid synthesis. This product has the potential to decrease the negative environmental impact of livestock production.

The composition of dietary nutrients, dietary components, and feeding systems are important for manipulating rumen microbial composition and fermentation. The study by Rehemujiang et al. found that replacing soybean meal with cottonseed meal or rapeseed meal in the diets of Hu sheep stimulated production of propionate in the rumen, improved the efficiency of energy utilization, and improved production performance of Hu sheep. Zhang et al. explored the effects of two feeding methods, indoor feeding and pasture grazing on the quality of black Tibetan sheep meat through metabolome and microbiome analyses. The results showed that indoor feeding of compound forage diets improved muscle water retention capacity, tenderness, and muscle color of black Tibetan sheep by regulating the metabolism of amino acids, lipids and carbohydrates in muscle tissues. In addition, indoor feeding practices positively affected the fatty acid and amino acid composition of muscle or meat. Hua et al. studied *in vitro* the effects of glucogenic and lipogenic diets on the structure of rumen bacteria and archaea and the *in vitro* rumen metabolome and gas production It was found that the lipogenic diet increased the proportion of cellulolytic bacteria, whereas with the glycogenic diets amylolytic bacteria were relatively more abundant. Bacteria with increased relative abundance in the glycogen diet played a role in the succinate pathway, resulting in higher propionate yield. Jiang et al. showed that supplementing yaks with 0.3% mixed isoacids increased the digestibility of neutral and acid detergent fiber and average daily weight gain, and changed the diversity of rumen bacteria.

New feed additives can promote rumen microbial fermentation and animal productivity. Xu et al. found that adding biochanin A (BCA), an isoflavone phytoestrogen, to the diet of dairy goats improved milk production performance, nitrogen metabolism and feed conversion efficiency. The authors found that BCA supplementation increased antioxidant performance and endocrine hormone levels, and altered composition of rumen microbiota, improved nitrogen metabolism by inhibiting degradation of amino acids, and increased the abundance of cellulolytic bacteria. This finding provides an effective nutritional means to improve the milk production performance of dairy goats. Tondini et al. explored how polyclonal antibodies produced against key rumen cellulolytic bacterial strains inhibited the growth of targeted strains in single culture. Their results show that polyclonal antibodies could be used to inhibit the growth of specific, undesirable rumen bacteria, and improve rumen fermentation patterns.

The host is also a driver for rumen microbiome changes. Wang et al. found that litter size had different effects on rumen microbiota community composition, rumen fermentation, and growth performance of goats. Their results show decreased growth performance and rumen concentration of total VFA, propionate, and butyrate, in triplet goats. Triplet goats have higher levels of *Prevotella* in their rumen and lower abundance in *Rikenellaceae RC9*, which can be related to growth performance of triplet goats.

Rumen development is important for young ruminants. Early consumption of solid feed can promote rumen growth and development in ruminants (Lin et al., 2019) and stimulate the development of rumen microbial communities (Liu et al., 2017). Huang et al. found that increasing the supply of milk replacer from 2 to 4% of lamb body weight increased weight gain, but reduced intake of starter solid feed and slowed down rumen development. High levels of milk replacer feeding increased rumen microbial diversity but reduced the relative abundance of carbohydrate-degrading bacteria. The results of Liu et al. showed that partially replacing alfalfa with oats in lamb diet promoted the production of beneficial fatty acids in *longissimus lumborum* muscle. A review by Du et al. reported the importance of using early intervention methods for the establishment of a healthy calf gastrointestinal microbiota. For example, oral probiotics, and fecal and rumen transplantation of microbiota, can be effective in alleviating calf diarrhea.

Ma et al. analyzed the differences in prokaryotic communities in lamb rumen fed granular TMR and bacterial communities in rumen contents and filtered rumen fluid. The results showed that the microbial DNA yield, bacterial diversity and abundance of fibrolytic bacteria and archaeal *Methanimicrococcus* in lamb rumen fluid were lower than those in mixed rumen content.

Urea is an important non-protein source of nitrogen for ruminants. In the rumen, ureolytic bacteria play a key role in urea nitrogen metabolism, however, the number of ureolytic bacteria isolated from the rumen is scarce. Zhong et al. isolated a novel ureolytic bacterium from cattle rumen and characterized its genome and function. The isolate is a new gram-positive strain of *Enterobacter hormaechei* named *E. hormaechei* Z129 that carries unique genes related to feed conversion, nitrogen dissimilation reduction, and lactose synthesis.

The present Research Topic highlights the interaction of rumen microbes with host and nutrient metabolism and animal production. It also provides innovative ideas for further research in this area informing practical management options to optimize ruminant production. The rumen microbiome not only breaks down ruminant feed, but also controls animal productivity and influences the quality of the final product. Future research should explore the molecular mechanisms by which the rumen microbiome regulates ruminant performance, with the aim of producing high-quality products that improve profitability and reduce environmental impact.

## Author contributions

QX: Writing—review and editing. EU: Writing—review and editing, Conceptualization. DM: Writing—review and editing, Conceptualization. SW: Writing—review and editing, Conceptualization. JL: Writing—review and editing, Conceptualization. WD: Writing—original draft. SZ: Writing—review and editing.
